# Food Fever in Children

**Published:** 1906-03-03

**Authors:** 


### FOOD FEVER IN CHILDREN.

Dr. Eustace Smith, under this title, describes an attack of fever which comes on suddenly, is accompanied by signs, more or less pronounced, of digestive disturbance, lasts in its acute form for several days, and may linger on in a modified degree for some weeks. The attacks may recur at frequent intervals, every month or so, and in these circumstances may seriously prejudice nutrition. The subjects of the complaint are usually neurotic children from three or four to ten or twelve years of age. The earliest symptom of the attack is headache, and with this is vomiting, looseness of the bowels, or other form of digestive disturbance. Suddenness of onset in a previously healthy child is one of the prominent features of these attacks. The temperature ranges from 100° to 105°, the tongue is dirty, percussion shows the stomach resonance to be extended upwards, the liver edge can be felt below the costal margin, and the urine is very acid and high-coloured. Unless proper treatment is adopted fever persists and tends to rise in the evening and also after food. After a few days it subsides, sometimes suddenly, with free sweating, sometimes gradually, but it may remain in a moderate degree for a week or more. If, on the other hand, the child at the very outset gets a good dose of calomel or blue-pill the temperature falls when the medicine has acted; it, however, rises again in the course of a few hours, and remains uncertain for some days, with a tendency to rise after food and in the evening. The patient is fretful, restless, and disinclined to take food, but makes no complaint of pain or other discomfort. In a highly neurotic child there may be convulsions. Vomiting is sometimes a prominent symptom, and probably many of the cases described as "periodic" or "cyclical" vomiting belong to this group; with the vomiting there may be abdominal pain. Should the attacks of fever, etc., be frequent, the child's general nutrition suffers, and pallor, loss of flesh, coldness of the feet, and other evidences of languid circulation are more or less pronounced. The cases are often exceedingly puzzling, and when recurrences are frequent and the general health suffers the suspicion of tuberculosis is apt to arise. The diagnosis depends on the history of the case—the intervals of comparatively good health and the invariable presence with the febrile attack of symptoms of gastro-intestinal disturbance.

The pathology of the condition is primarily an acute gastric catarrh due to chill, whilst the later fever is probably due to absorption of injurious products from the bowel.

To prevent and cut short the attacks a careful diet is of first importance. Not only excess of carbohydrates, but all articles of food capable of unhealthy fermentation, must be forbidden. Thus not only sweets and the ordinary farinaceous foods come under condemnation, but also milk itself. Acids as in fruits, lemonade, etc., must also be excluded, as they tend to promote fermentation. The most suitable diet in these cases is mutton, poultry, white fish, well-boiled green vegetables, and eggs. Butter may be given with stale bread, toast, and rusks. Salted food, as bacon, ham, and tongue, and anchovy, bloater and shrimp pastes, are good; the latter make good substitutes for marmalade and jam. The dieting, to be successful, must be thorough; a very small quantity of unsuitable food may be sufficient to maintain or revive unhealthy fermenting processes.

To prevent further attacks it is also necessary to take steps to avoid the recurrence of gastric catarrh. Hence the child should have abundance of outdoor exercise, and should wear sufficient clothing, more particularly on his feet and legs. Long woollen stockings should be worn indoors as well as outdoors, and in summer equally with winter; indeed, it is in the former season that rapid changes of temperature afford special opportunities for chill. Beyond fresh air and clothing the whole routine of the child's life should be scrutinised with a view to detect any ill-advised procedure. And when the attacks have been numerous it must be remembered that the child is much more susceptible to chill than is an ordinary child, and hence special precautions and care are demanded.

